# Sol–Gel-Derived Vinyltrimethoxysilane (VTMS)/Tetraetoxysilane (TEOS) Hybrid Coatings on Titanium Materials for Use in Medical Applications

**DOI:** 10.3390/ma18102273

**Published:** 2025-05-14

**Authors:** Oliwia Kierat, Agata Dudek

**Affiliations:** Department of Material Engineering, Faculty of Production Engineering and Materials Technology, Czestochowa University of Technology, Aleja Armii Krajowej 19, 42-200 Czestochowa, Poland

**Keywords:** Ti Gr2, Ti6Al4V, Ti13Nb13Zr, VTMS, TEOS, sol–gel, silane coatings

## Abstract

Hybrid silane-based coatings were developed via the sol–gel process using two precursors, vinyltrimethoxysilane (VTMS) and tetraethoxysilane (TEOS), and subsequently deposited onto three titanium-based substrates: commercially pure titanium Grade 2, Ti6Al4V, and Ti13Nb13Zr. Comprehensive physicochemical characterization was performed, including microstructural (optical and SEM), topographical (3D roughness), spectroscopic (FTIR), and electrochemical (potentiodynamic) analyses. The coatings were continuous, transparent, smooth, and exhibited high gloss with no visible cracks or surface defects. Surface roughness (Sa ≈ 0.3 μm) was consistent across all samples and remained unaffected by both the VTMS to TEOS ratio and the substrate type. Coating thickness ranged from 8 to 15 μm, as confirmed by both digital microscopy and thickness gauge measurements. All coatings demonstrated strong adhesion to the substrates. FTIR analysis confirmed the presence of key functional groups, such as CH_2_, C=C, C–H, Si–O–Si, Si–OH, Si–O–Ti, CH=CH_2_, and O–Si–O, regardless of the substrate type. Electrochemical tests in Ringer’s solution showed excellent corrosion resistance, particularly for coatings with a VTMS to TEOS ratio of 1:1. Post-corrosion imaging confirmed the integrity of the coatings and their effectiveness as protective barriers in simulated physiological environments. These findings support the potential of VTMS/TEOS sol–gel coating as a surface modification strategy for biomedical titanium implants.

## 1. Introduction

The dynamic development of surface engineering has promoted the implementation of hybrid materials capable of effectively combining chemically incompatible phases and forming functional protective coatings. Organofunctional silanes—organosilicon compounds—deserve particular attention due to their chemical structure, which enables simultaneous interaction with inorganic materials such as metals and glass, as well as with organic coatings, resins, or varnishes. Their ability to form durable chemical bonds between these two classes of materials makes them key components in modern protective and adhesive systems [1,2].

Silanes, generally described by the formula R_n_SiX_4−n_, where R represents an organic group and X represents a hydrolyzable group (most commonly alkoxy), are referred to as molecular adhesion promoters. Upon hydrolysis of the X groups and their subsequent condensation, silanols are formed, which then bond to inorganic surfaces through the formation of Si–O–M bonds (where M is a metal atom or its oxide), while simultaneously enabling interaction with the organic layer via the R group. This dual reactivity underlies their high efficiency as interfacial layers in coating systems [3,4,5,6].

A wide range of silanes is used in both industrial and laboratory applications—from hydrophobic methyltriethoxysilanes, through epoxysilanes, aminosilanes, and mercaptosilanes, to bifunctional compounds. Vinylsilanes (e.g., vinyltriethoxysilane, VTMS) and tetraalkoxysilanes, including tetraethoxysilane (TEOS), which is one of the most commonly used inorganic precursors, are of particular importance. The choice of a specific silane depends on the type of substrate, the target application properties of the coating (such as corrosion resistance, mechanical strength, and barrier performance), and its chemical compatibility with the base material [7,8,9].

The deposition of silanes onto surfaces can be carried out using various techniques, among which one of the most commonly employed is dip-coating. This method involves immersing the substrate in a water–alcohol solution containing an appropriate catalyst (acidic or basic). In applications requiring precise control over film thickness—such as microelectronics or surface engineering—the spin-coating technique is widely used, enabling the deposition of thin films at the nanometer scale. Alternatively, for industrial-scale applications, spray-coating methods are also utilized.

For more advanced inorganic or hybrid coatings, particularly those with a three-dimensional structure, the sol–gel technique is employed. This approach is based on the hydrolysis and condensation of alkoxysilane precursors (e.g., TEOS), leading to the formation of a cross-linked polysiloxane matrix. For applications demanding exceptionally high surface cleanliness, such as precision optics or semiconductors, vapor-phase deposition techniques (CVD) and preliminary plasma surface modifications are also used [10].

Due to their broad spectrum of physicochemical properties and the possibility of chemical modification, silanes have found applications across numerous industrial sectors. In the automotive industry, they are used as primer layers to enhance the adhesion of paints and composite materials. In electronics, they serve as hydrophobic, dielectric, and barrier coatings. In civil engineering, silanes are employed in concrete impregnation and protecting structures against moisture. They also play significant roles in the dyeing, printing, textile, and biomedical industries. Depending on the type of silane used, their functions may vary—from improving adhesion, providing corrosion protection, and imparting hydrophobicity, to modifying the surface properties of materials [5,11].

Despite their common silicon–oxygen-based backbone structure, the physicochemical properties of individual silanes can vary significantly depending on the type of functional groups attached. It is the diversity of substituents—both organic and inorganic—that determines their application-specific properties and allows tailoring to particular substrate types and service conditions. As a result, silanes constitute a class of compounds with high adaptive potential, capable of selective chemical and physical interaction with a wide range of materials.

**Figure 1:**
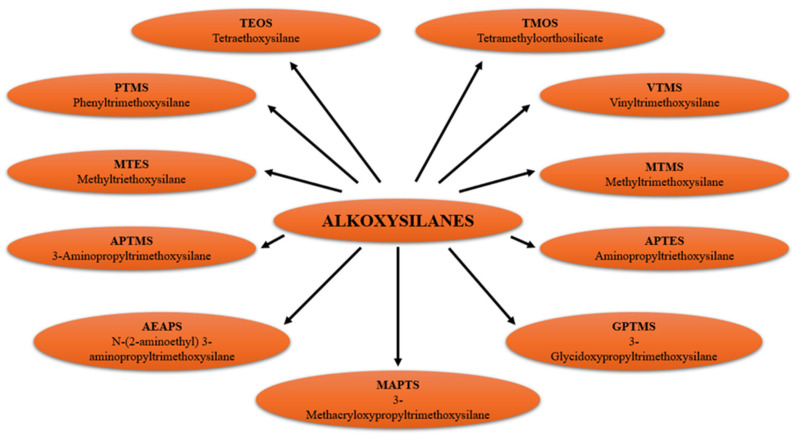
Overview of the most commonly used alkoxysilanes as precursors for organic–inorganic hybrid materials. Compiled based on [9].

In practice, the most commonly used group of compounds are alkoxysilanes—molecules containing hydrolyzable –OR groups (typically methoxy or ethoxy), which undergo condensation to form a three-dimensional polysiloxane network. Figure 1 presents a selection of representative alkoxysilanes used as precursors for organic–inorganic hybrid materials [5,12,13,14,15,16,17].

**Types of Alkoxysilanes: Properties and Application Relevance**

- Tetraalkoxysilanes (e.g., TEOS—tetraethoxysilane) do not contain organic groups, which makes them suitable for the synthesis of pure silica-based coatings with high hardness, chemical stability, and excellent barrier properties. Due to the absence of functional organic groups, their application is limited to passive structures without the potential for further chemical modification.
- Monofunctional silanes, such as MTES (methyltriethoxysilane) or DMDES (dimethyldiethoxysilane), contain hydrophobic groups that influence the wettability and flexibility of the resulting coatings. They are mainly used as surface property modifiers, serving barrier and protective functions.
- Vinylsilanes (e.g., VTMS—vinyltriethoxysilane) possess vinyl groups capable of undergoing addition or polymerization reactions, enabling their permanent chemical anchoring into organic structures such as acrylic resins. They act as effective coupling agents between organic and inorganic components in hybrid materials.
- Epoxysilanes, such as GPTMS (3-glycidyloxypropyltrimethoxysilane), exhibit high reactivity toward amines and hydroxyl groups, making them commonly used for the cross-linking of epoxy resins and in functionalized systems that combine mechanical requirements with bioactivity.
- Aminosilanes (e.g., γ-APS—3-aminopropyltrimethoxysilane) contain amino groups capable of electrostatic and covalent interactions with proteins, peptides, and nucleic acids, which makes them useful in bioengineering, medical applications, and diagnostic technologies.
- Sulfidosilanes and mercaptosilanes contain thiol (–SH) groups that exhibit strong affinity for metallic surfaces, especially silver and gold. They are used, among other uses, in sensor technologies, antibacterial coatings, and in the surface chemistry of noble metal-based materials.

Moreover, silanes can be combined into hybrid systems, in which a single matrix contains both functional groups (e.g., vinyl, epoxy) and alkoxysilane moieties responsible for forming a three-dimensional siloxane network. Such combinations—like TEOS with GPTMS—enable the fabrication of coatings with a desirable combination of properties, including high mechanical resistance, bioactivity, and structural flexibility [18,19,20,21].

**Mechanism of Silane Hydrolysis and Condensation in the Sol–Gel Process**

The sol–gel process is a low-temperature chemical technique that enables the synthesis of cross-linked silica-based and organic–inorganic hybrid materials with controlled structures. In the case of alkoxysilanes such as tetraethoxysilane (TEOS) or vinyltriethoxysilane (VTMS), the process consists of two main stages: hydrolysis and condensation [22,23,24].

During the hydrolysis stage, silane molecules react with water, in which the alkoxy groups (–OR) are progressively replaced by hydroxyl groups (–OH). This reaction leads to the formation of silanol compounds, which serve as intermediates for subsequent condensation steps:

Si(OR)_4_ + H_2_O → Si(OR)_3_(OH) + ROH

Si(OR)_3_(OH) + H_2_O → … → Si(OH)_4_

Condensation involves the coupling of the resulting silanol or silanol–alkoxy groups, leading to the formation of siloxane bonds (Si–O–Si) with the release of either water or alcohol molecules:

Si–OH + Si–OH → Si–O–Si + H_2_O

Si–OH + Si–OR → Si–O–Si + ROH

In the subsequent stages of condensation, oligomers are formed and undergo further cross-linking, leading to the development of a three-dimensional gel structure with an immobilized liquid phase entrapped within the siloxane matrix.

Key sol–gel process parameters—such as the type of catalyst (acidic or basic), water-to-silane molar ratio, solvent, temperature, and the presence of additives—allow precise control over the morphology, cross-linking rate, and coating properties.

The mechanisms of hydrolysis and condensation reactions are highly dependent on pH and the nature of the reaction environment. Under acidic conditions, the cross-linking process proceeds predominantly through the formation of linear oligomeric structures, whereas basic environments promote the rapid formation of highly branched, three-dimensional networks. These differences directly influence the morphology, degree of cross-linking, and porosity of the resulting sol–gel-derived coatings [21,25,26].

Among the wide range of available silanes, vinyltriethoxysilane (VTMS) and tetraethoxysilane (TEOS) are of particular significance due to their complementary properties, despite differences in their chemical structures. VTMS, owing to the presence of a reactive vinyl group, can participate in cross-linking reactions with organic components, making it especially useful in hybrid formulations that require enhanced chemical and mechanical resistance. TEOS, on the other hand, as a classical tetraalkoxysilane, acts as a silica precursor in sol–gel processes, enabling the formation of hard, transparent coatings with high chemical stability and excellent barrier properties.

The combination of these two compounds within a single system allows for the development of protective coatings that integrate the flexibility and functionality of VTMS with the durability and three-dimensional cross-linked network provided by TEOS. This synergy forms the basis for designing advanced anticorrosion systems intended for application on metallic surfaces [27,28,29].

In the literature, several studies can be found on sol–gel coatings containing VTMS and TEOS; however, their applications have so far been limited to technical materials rather than biomedical ones. For example, Álvarez et al. [30] developed sol–gel coatings based on VTMS and TEOS that were deposited on tinplate substrates. The authors investigated the effect of the molar ratio of the precursors on the functional properties of the material, indicating that the best protective and structural performance was achieved for systems with VT50 (50% VTMS:50% TEOS) and VT25 (25% VTMS:75% TEOS) compositions.

During the synthesis process, 2-propanol was used as the solvent and nitric acid as the acidifying catalyst. The aim of the coatings was to reduce corrosion processes in packaging materials intended for food contact. The hybrid silane system served as an alternative to conventional lacquer coatings, offering an effective protective barrier. The presence of these layers on the tinplate surface effectively limited the access of corrosive media and prevented the migration of corrosion products into the interior of the packaging, thereby minimizing the risk of contamination and potential health hazards.

The biomedical applications of metallic materials are primarily limited by the phenomenon of corrosion. Corrosion of metal implants placed inside the human body leads to the release of substances that are harmful to the organism, thereby causing inflammation and, ultimately, implant rejection [31]. The implanted material should be biocompatible, i.e., it should not have a negative effect on the host organism. A common method of enhancing the biocompatibility of metallic materials is the application of protective surface coatings designed to prevent implant failure. In the present study, a novel application of organic–inorganic silane-based coatings composed of VTMS and TEOS is proposed in the context of biomaterial engineering, with particular emphasis on their potential use in implantology. One of the major clinical challenges associated with metallic implants remains corrosion, the products of which may induce inflammatory responses, leading to implant rejection, and ultimately necessitate revision surgery. Therefore, the development of durable, biocompatible protective systems is of significant practical importance.

In this work, titanium and its alloys were used as substrates for coating deposition. Compared to stainless steel, titanium-based biomaterials exhibit excellent biocompatibility and promote osseointegration, which justifies their widespread clinical use. Among various approaches to titanium surface modification, oxide coatings—particularly those based on stoichiometric and non-stoichiometric titanium oxides such as TiO_2_, TiO_2−x_, and TinO_2n−1_—have been widely studied for their corrosion protection, bioactivity, and sensor functionality. As reviewed by Ramanavicius et al. [32], these systems highlight the adaptability of titanium-based surfaces to a range of advanced applications. In the present study, we propose a distinct strategy based on hybrid silane coatings derived from vinyltrimethoxysilane (VTMS) and tetraethoxysilane (TEOS) to explore their potential in biomedical contexts. The coatings were obtained via the sol–gel method, using VTMS and TEOS solutions as precursors, ethanol as the solvent, and acetic acid as the hydrolysis catalyst.

Three types of titanium substrates were selected for the study: (a) commercially pure titanium Grade 2, (b) Ti6Al4V alloy, and (c) Ti13Nb13Zr alloy. The selection criteria included both their established medical applications and their differences in chemical composition and mechanical properties, which may influence the efficiency of the sol–gel process and the quality of the resulting layers. Titanium Grade 2 is characterized by good corrosion resistance and biocompatibility, though its mechanical strength is limited. Ti6Al4V alloy, widely used in implantology, combines high mechanical strength with acceptable chemical stability; however, the potential release of aluminum and vanadium ions may present limitations. Ti13Nb13Zr alloy, free of cytotoxic elements, exhibits a low elastic modulus and excellent biocompatibility, making it a modern alternative to traditional titanium alloys.

The use of three distinct substrate materials enables a comprehensive assessment of the effectiveness of sol–gel coatings in the context of their potential applications in biomaterial engineering, as well as an analysis of the influence of substrate properties on the deposition process and final characteristics of the protective layer.

In contrast to previous studies, such as the work of Álvarez et al. [30], in which nitric acid was used as a catalyst and the coatings were synthesized on steel substrates, the present study introduces a novel approach in terms of both substrate selection and the chemical conditions of sol–gel synthesis. For the first time, a combination of VTMS and TEOS precursors was employed in the presence of acetic acid as a mild acid catalyst to produce hybrid sol–gel coatings deposited on biocompatible titanium-based substrates. This specific configuration, which also considers the differing chemical and mechanical properties of the titanium alloys used, has not been previously reported in the literature in the context of biomedical applications.

Such a system design not only enables the formation of coatings with good adhesion and corrosion resistance but also provides a foundation for further research into the bioactivity and cytocompatibility of these layers, particularly regarding their clinical applicability in implant engineering.

These hybrid coatings may be applicable to a wide range of titanium-based implants, such as dental implants, hip and knee endoprostheses, bone screws, and spinal fixation systems, where long-term stability and corrosion resistance are crucial. Unlike traditional oxide coatings or polymer films, VTMS/TEOS-based sol–gel layers offer a tunable chemical structure, allowing not only for effective barrier properties but also potential for further functionalization with bioactive agents such as antibiotics, growth factors, or osteoinductive peptides. This opens up prospects for multifunctional implant coatings that simultaneously protect the substrate and actively support tissue integration and healing.

Therefore, this study aims to evaluate the protective potential of VTMS/TEOS-derived coatings on titanium-based substrates used in implantology, focusing on their corrosion resistance in simulated physiological conditions. The results are expected to support the development of multifunctional, durable surface treatments for future biomedical applications.

## 2. Materials and Methods

The coatings were applied onto three types of substrates: Ti Grade 2, Ti6Al4V, and Ti13Nb13Zr. The chemical compositions of the alloys, as provided in the technical documentation from the manufacturer, are summarized in Table 1. The samples were prepared in the form of cylindrical discs with a diameter of 5 mm, embedded in holders made of polymethyl methacrylate (PMMA) using epoxy resin. Prior to coating deposition, the substrate surfaces were subjected to wet mechanical polishing using silicon carbide abrasive papers with grit sizes up to P2000. The samples were then rinsed with distilled water and degreased with acetone.

A uniform surface preparation procedure was applied to all three materials in order to ensure reproducible sol–gel processing conditions and to eliminate the influence of local surface topography variations on the coating properties and experimental results.

The coatings were prepared using the following analytical grade (≥99%) reagents: (i) vinyltrimethoxysilane (VTMS) from Sigma Aldrich (St. Louis, MO, USA), (ii) tetraethoxysilane (TEOS) from Sigma Aldrich, (iii) ethanol (EtOH) from Chempur (Piekary Śląskie, Poland), and (iv) acetic acid (AcOH) from Chempur.

To obtain four different coatings with varying VTMS to TEOS ratios, four precursor solutions were prepared with the following volume ratios:

1. VTMS:TEOS:EtOH:AcOH:H_2_O = 0.6:0.0:0.2:0.06:0.14,
  applied to three different substrates:
  (a) Ti Grade 2—coating 1a
  (b) Ti6Al4V—coating 1b
  (c) Ti13Nb13Zr—coating 1c

2. VTMS:TEOS:EtOH:AcOH:H_2_O = 0.5:0.1:0.2:0.06:0.14,
  applied to three different substrates:
  (d) Ti Grade 2—coating 2a
  (e) Ti6Al4V—coating 2b
  (f) Ti13Nb13Zr—coating 2c

3. VTMS:TEOS:EtOH:AcOH:H_2_O = 0.4:0.2:0.2:0.06:0.14,
  applied to three different substrates:
  (g) Ti Grade 2—coating 3a
  (h) Ti6Al4V—coating 3b
  (i) Ti13Nb13Zr—coating 3c

4. VTMS:TEOS:EtOH:AcOH:H_2_O = 0.3:0.3:0.2:0.06:0.14,
  applied to three different substrates:
  (j) Ti Grade 2—coating 4a
  (k) Ti6Al4V—coating 4b
  (l) Ti13Nb13Zr—coating 4c

The reaction solutions were mixed using a magnetic stirrer for 24 to 48 h to ensure complete hydrolysis and initiate the condensation of the silane precursors. The samples were then immersed in the prepared solution for 20 min, and after immersion, they were withdrawn vertically at a constant speed of 2 mm·s^−1^. Excess solution was gently removed using filter paper. The coated samples were dried under laboratory conditions (temperature 23 °C, relative humidity 45%) for 24 to 72 h. No additional thermal treatment was applied—the condensation and gelation processes proceeded under static conditions inside a desiccator.

Microstructural surface analysis and roughness measurements were performed using a KEYENCE VHX-7000 digital microscope (Keyence, Mechelen, Belgium). A three-dimensional surface topography analysis was conducted, including the following roughness parameters:

- Sa—arithmetic mean deviation of surface heights from the mean plane,
- Sz—maximum height of the surface,
- Sq—root mean square (RMS) height deviation,
- Ssk—skewness (asymmetry of the height distribution),
- Sku—kurtosis (peakedness or sharpness of the height distribution),
- Sp—height of the highest peak,
- Sv—depth of the deepest valley.

Additional microstructural analysis was performed using a scanning electron microscope (SEM), JEOL JSM-6610LV (JEOL Ltd., Tokyo, Japan), including energy-dispersive X-ray spectroscopy (EDX) elemental mapping to assess the chemical composition and distribution of elements in the cross-sectional areas of the coatings. Coating adhesion to the substrate was evaluated using the Scotch™ tape test (Scotch™ Brand, St. Paul, MN, USA) according to ASTM D3359 [33]. Coating thickness was measured using a KEYENCE VHX-7000 digital microscope and a Testan DT-10 AN 120 157 thickness gauge (Anticorr, Gdańsk, Poland). For each coating variant, the thickness was measured at ten different regions on the sample surface. The values reported in Section 3 represent the arithmetic mean thickness (μm) along with the standard deviation (±SD) calculated from these ten measurements. This approach was applied consistently across all coating–substrate combinations. Coating structure was characterized using an IRAffinity-1S FTIR spectrophotometer (SHIMADZU, Kyoto, Japan).

Corrosion testing was carried out using a CH Instruments 440A electrochemical workstation (CH Instruments, Austin, TX, USA) in a three-electrode setup, with a saturated calomel electrode (SCE, +0.244 V vs. SHE at 25 °C) as the reference electrode, a platinum electrode as the counter electrode, and the coated substrate as the working electrode. Potentiodynamic polarization curves were recorded over a potential range from −1.5 V to +3.0 V vs. SCE at a scan rate of 0.01 V·s^−1^. Corrosion resistance tests were performed in Ringer’s solution with the following composition: NaCl—8.6 g/dm^3^, KCl—0.3 g/dm^3^, and CaCl_2_·2H_2_O—0.333 g/dm^3^ [34].

For each coating, three independent measurements were performed under identical conditions, and the results presented in this article represent mean values (±0.012–0.018 V). Based on the recorded potentiodynamic curves, polarization resistance plots were constructed for each sample, showing the relationship ∆U = f(∆i) in the vicinity of the corrosion potential (±25 mV). These plots were used to calculate polarization resistance values (Rp ± SD) for both uncoated substrates and coated samples. According to the Stern–Hoar equation, the external current density is a linear function of potential, and the slope of the corresponding straight lines represents the measure of polarization resistance [35].

## 3. Results and Discussion

### 3.1. Microstructure and Surface Roughness of the Coatings

**Figure 2:**
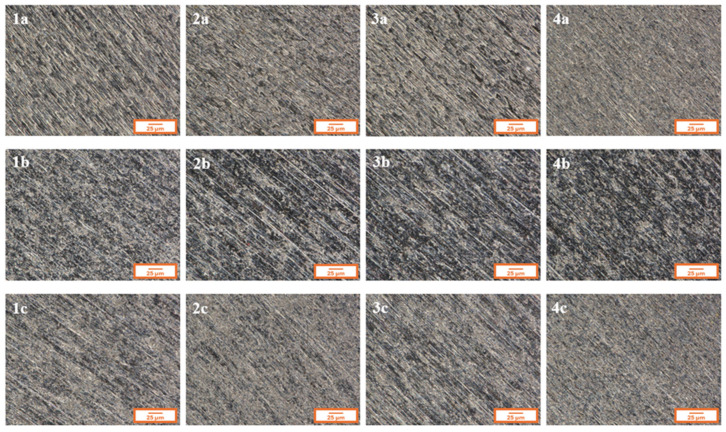
Microstructures of individual coatings recorded using a digital microscope.

**Figure 3:**
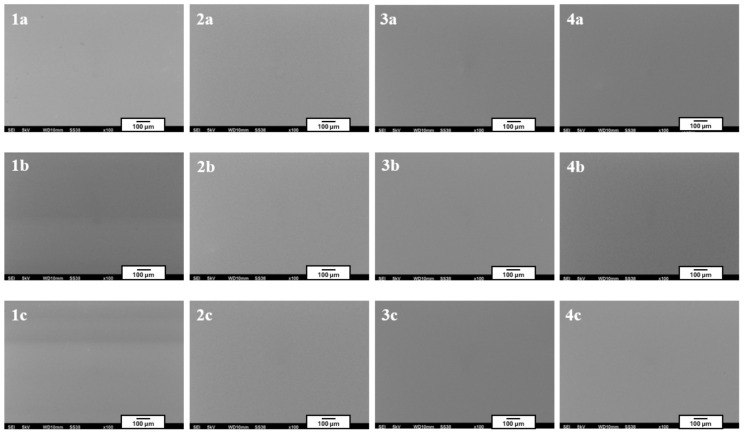
Microstructures of individual coatings recorded using a scanning electron microscope (images taken at 100× magnification).

Figure 2 presents the microstructure of all analyzed coatings obtained using digital microscopy, while Figure 3 shows the coating microstructures acquired using scanning electron microscopy (SEM).

Table 2, Table 3 and Table 4 present the results of 3D surface roughness analysis for the coatings deposited on the three titanium substrates (Ti Grade 2, Ti6Al4V, and Ti13Nb13Zr). The values of key topographic parameters—previously defined in the Section 2—allowed for comparative evaluation of surface morphology and uniformity across different precursor ratios and substrate types.

In Figure 2, for each of the analyzed coatings, the underlying substrate surface structure is clearly visible, indicating high transparency of the deposited layers. Both the digital microscopy images (Figure 2) and the scanning electron microscopy images (SEM, Figure 3) confirmed that the fabricated coatings exhibited a smooth, uniform surface morphology, a pronounced gloss, and no visible defects such as cracks, discontinuities, or delamination.

The microscopic observations also confirmed good adhesion of the coatings to the substrate—the layers evenly covered the entire examined surface without any visible interruptions in continuity.

The surface roughness analysis, presented in Table 2, Table 3 and Table 4, showed that all coatings, regardless of precursor ratio or titanium alloy type, exhibited uniform topography with Sa values around 0.3 μm. For comparison, the Sa value of the pre-treated substrates—after polishing (grit P2000), rinsing with distilled water, and degreasing with acetone—was approximately 0.4 μm. Thus, the application of sol–gel coatings effectively reduced the surface roughness, which is beneficial for corrosion protection by minimizing potential sites for corrosion initiation and enhancing the material’s long-term durability.

In addition to the influence of precursor composition, the effect of substrate alloy composition was also considered. The use of three different titanium alloys—commercially pure Ti Grade 2, Ti6Al4V, and Ti13Nb13Zr—enabled a comprehensive evaluation of how substrate composition affects the properties of sol–gel-derived coatings. Variations in alloying elements, such as aluminum and vanadium (in Ti6Al4V) or niobium and zirconium (in Ti13Nb13Zr), may influence both the surface energy and the chemical affinity of the substrate toward sol–gel precursors. Despite these differences, all coatings exhibited comparable surface roughness, uniform morphology, and strong adhesion, confirming the broad applicability of the VTMS/TEOS system across various titanium-based substrates. Minor deviations in parameters such as Sa, Sz, or Rp may reflect the impact of specific surface characteristics—such as the reactivity of native oxide layers or microtexture—on the coating formation process and its final properties.

To expand the scope of the study, additional sol–gel formulations were developed with increased content of the inorganic component TEOS. The composition of the first solution series was VTMS:TEOS:EtOH:AcOH:H_2_O = 0.2:0.4:0.2:0.06:0.14, and it was applied to three different substrates:

Ti Grade 2—sample labeled 5a,
Ti6Al4V—sample 5b,
Ti13Nb13Zr—sample 5c.

The second formulation (0.1:0.5:0.2:0.06:0.14) was applied to samples 6a (Ti Grade 2), 6b (Ti6Al4V), and 6c (Ti13Nb13Zr), respectively. In the third series, VTMS was completely eliminated from the composition (0.0:0.6:0.2:0.06:0.14), resulting in a purely inorganic sol–gel matrix. The corresponding samples were labeled 7a, 7b, and 7c, depending on the substrate used.

**Figure 4:**
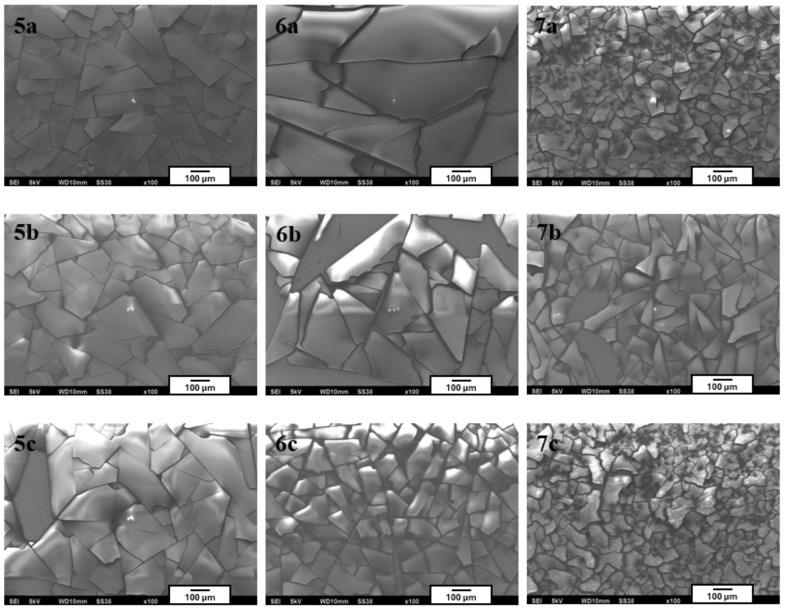
Microstructures of individual coatings recorded using a scanning electron microscope (images taken at 100× magnification).

Unfortunately, all coatings from this series exhibited high brittleness and poor adhesion to the substrate surface. Numerous cracks and delamination phenomena were observed, visible both to the naked eye and under the microscope (Figure 4). Due to their unsatisfactory mechanical properties and lack of coating continuity, these coatings were excluded from further analysis.

The observed brittleness and tendency toward the delamination of coatings with increased TEOS content may have been directly related to the intensified condensation processes of the inorganic polysiloxane network occurring during sol–gel synthesis. The high proportion of TEOS—an entirely inorganic precursor lacking functional groups capable of stress compensation—promoted the formation of highly cross-linked, rigid structures with limited flexibility. As a result, these layers were particularly susceptible to shrinkage and thermal stresses that arise during the drying and aging phases of the coating.

The absence of organic components—such as vinyl groups (e.g., from VTMS), epoxy groups, or other cross-linking modifiers—prevented effective stress distribution within the matrix, which can lead to the initiation of microcracks and localized adhesive failures. An additional destabilizing factor may have been the mismatch in thermal expansion coefficients between the inorganic coating layer and the metallic substrate. This phenomenon may generate additional interfacial stresses, especially during the transition from the gel phase to the dry coating, ultimately resulting in delamination and reduced coating integrity.

Similar degradation mechanisms of sol–gel coatings resulting from excessive cross-linking of the inorganic phase and the absence of flexibilizing agents have been reported in the literature. As indicated by previous studies [36,37,38,39,40], excessive condensation of TEOS in systems lacking organic groups leads to a reduction in coating flexibility and adhesion, thereby increasing the risk of cracking, embrittlement, and delamination from the substrate.

### 3.2. Coating Adhesion to the Substrate

The adhesion of the coatings to metallic surfaces was evaluated using the Scotch™ tape test in accordance with Procedure B defined in the ASTM D3359 standard, which allows for qualitative classification of coating adhesion based on the extent of detachment. Following the test, it was found that the area of coating removed by the tape did not exceed 5% of the total surface within the designated test area for any of the samples. This low level of detachment indicated very good coating integrity with the substrate and qualified the tested coatings in category 4B, which corresponds to high adhesion according to the standard.

This result is particularly important in the context of biomedical applications, where the stability of the protective layer in contact with physiological solutions is crucial for maintaining implant functionality and long-term durability. The high adhesion of the coatings obtained under sol–gel processing conditions—without additional thermal curing—demonstrated the effectiveness of the VTMS/TEOS system and the well-optimized surface preparation procedure used prior to coating deposition.

### 3.3. Coating Thicknesses

The average thickness values of all analyzed coatings, along with estimated measurement uncertainties, were determined using the Testan DT-10 AN 120 157 thickness gauge (Anticorr, Gdańsk, Poland) and are summarized in Table 5. Analysis of the results indicated that the coating thickness values for individual samples did not differ significantly—remaining within the range of measurement uncertainty—which suggested high reproducibility of the coating deposition process under the applied experimental conditions.

**Figure 5:**
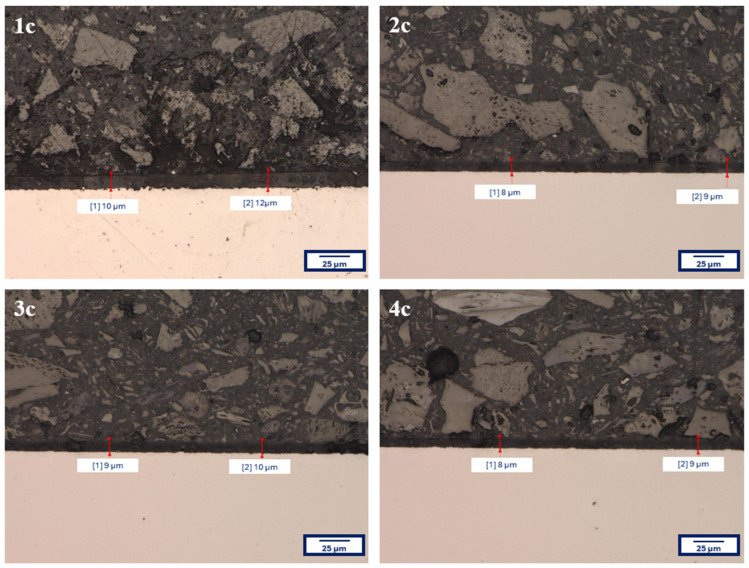
Cross-sectional images of the coatings on the Ti13Nb13Zr titanium alloy substrate with indicated thicknesses of individual coatings.

For verification purposes, additional thickness measurements were performed using a KEYENCE VHX-7000 digital microscope (Keyence, Mechelen, Belgium). The procedure involved capturing cross-sectional images of the samples and directly measuring the coating thickness at magnification. Figure 5 presents the measurement results for a selected example—coatings deposited on the Ti13Nb13Zr alloy.

The obtained coating thickness values, regardless of the measurement method used, showed good agreement, confirming the reliability of the results and the uniformity of the synthesis process.

### 3.4. Coating Structure Characterization

**Figure 6:**
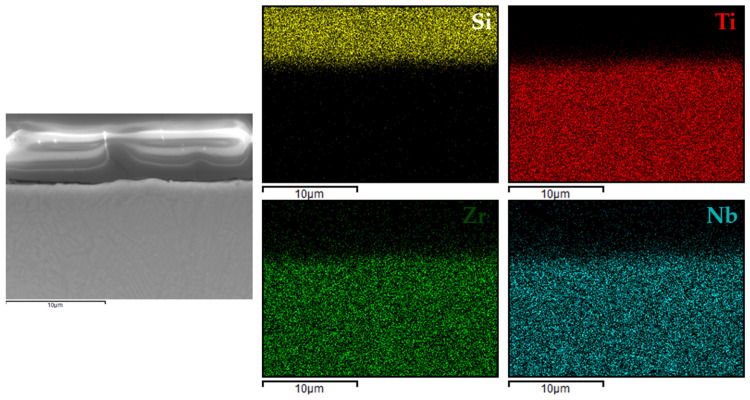
EDX elemental mapping for 1c coating.

**Figure 7:**
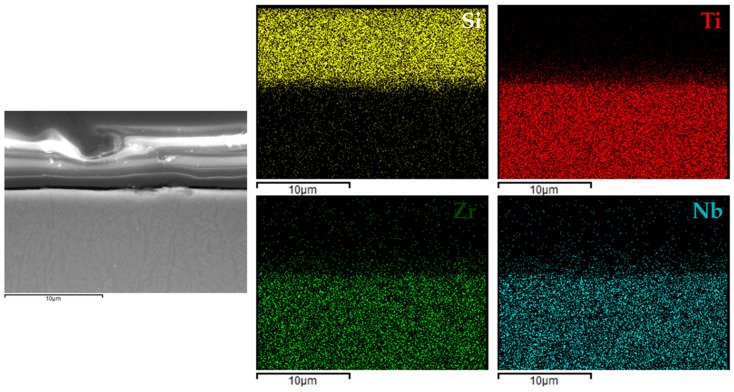
EDX elemental mapping for 2c coating.

**Figure 8:**
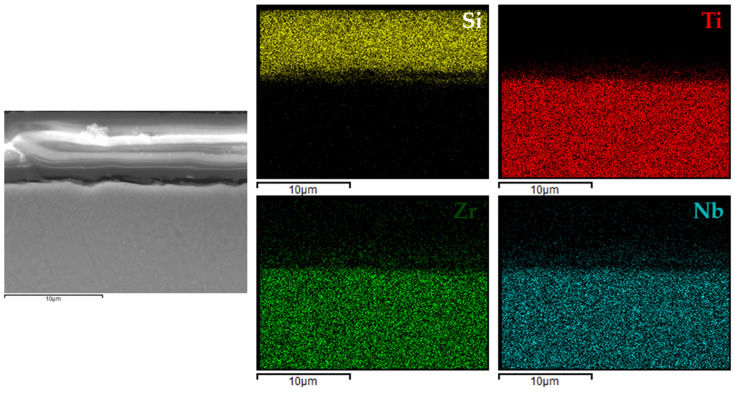
EDX elemental mapping for 3c coating.

**Figure 9:**
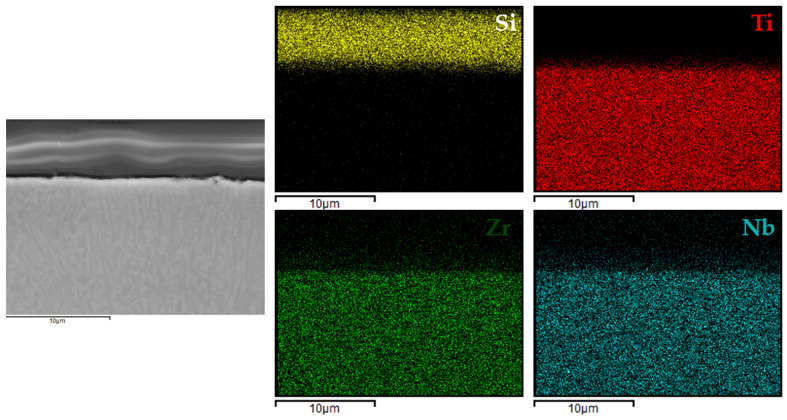
EDX elemental mapping for 4c coating.

Figure 6, Figure 7, Figure 8 and Figure 9 present the EDX elemental mapping results for samples 1c, 2c, 3c, and 4c deposited on the Ti13Nb13Zr titanium alloy substrate.

**Figure 10:**
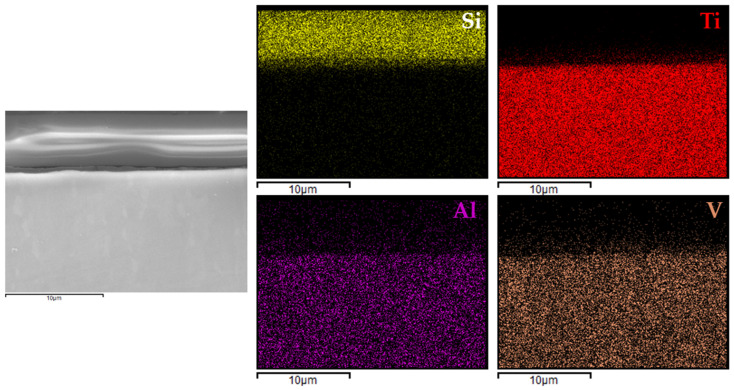
EDX elemental mapping for 4b coating.

**Figure 11:**
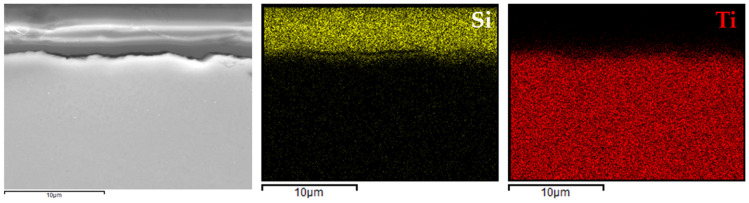
EDX elemental mapping for 4a coating.

For comparison, analogous analyses were also performed for the most promising coating (variant 4) applied to Ti6Al4V (Figure 10) and Ti Grade 2 (Figure 11) substrates.

The EDX elemental mapping confirmed the successful deposition of silane-based coatings on all tested titanium substrates. For the Ti13Nb13Zr alloy (Figure 6, Figure 7, Figure 8 and Figure 9), a uniform distribution of silicon was observed across the cross-section, indicating even surface coverage and good integration of the sol–gel-derived layer with the substrate.

In the case of variant 4 coatings applied to the Ti6Al4V alloy (Figure 10), silicon was clearly localized within the coating region, while aluminum and vanadium signals were confined to the substrate. A similar distribution was observed for the coating on titanium Grade 2 (Figure 11), where silicon was evenly dispersed within the coating layer and the titanium signal originated solely from the substrate.

These findings indicated that the applied process resulted in continuous, homogeneous, and well-adhered protective layers, regardless of the type and chemical composition of the titanium substrate. This confirmed the versatility of the VTMS/TEOS system in forming coherent protective coatings on materials intended for biomedical applications.

**Figure 12:**
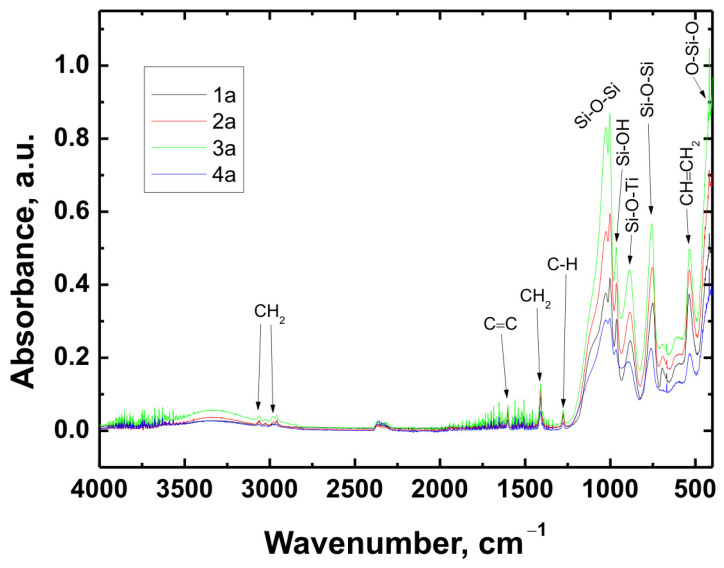
FTIR spectra obtained for coatings 1a, 2a, 3a, and 4a deposited on the Ti Grade 2 substrate.

**Figure 13:**
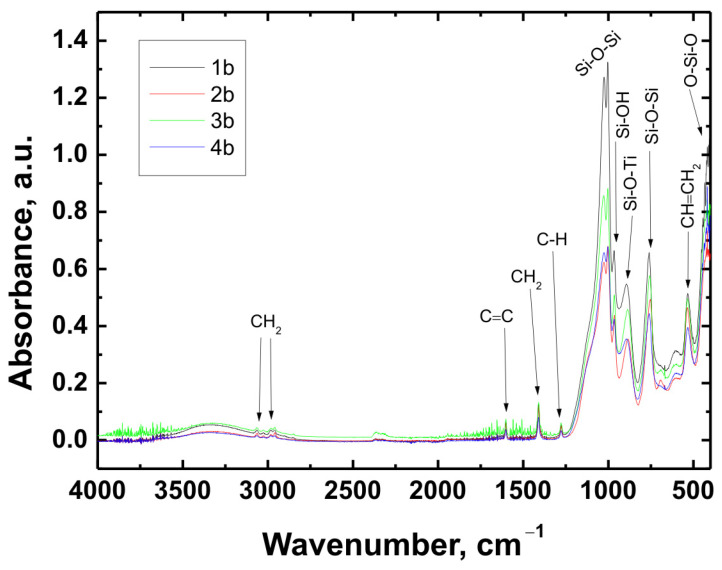
FTIR spectra obtained for coatings 1b, 2b, 3b, and 4b deposited on the Ti6Al4V substrate.

**Figure 14:**
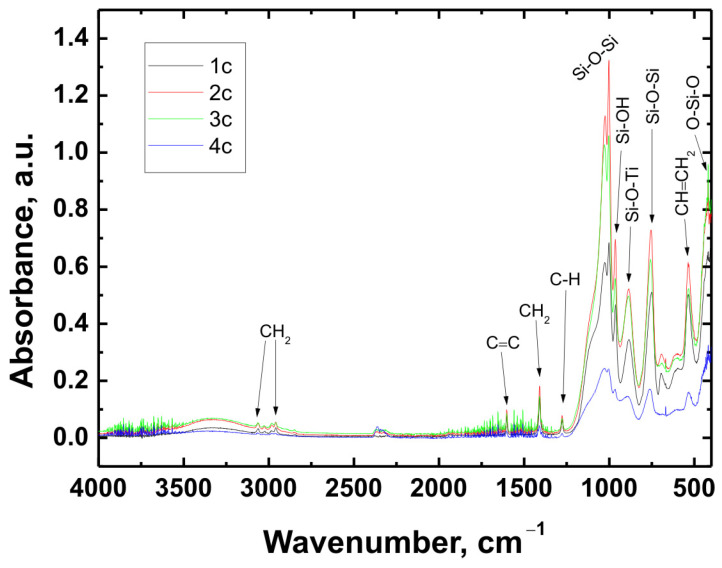
FTIR spectra obtained for coatings 1c, 2c, 3c, and 4c deposited on the Ti13Nb13Zr substrate.

The chemical structure of all coating variants deposited on the three types of substrates (Ti Grade 2, Ti6Al4V, and Ti13Nb13Zr) was analyzed using Fourier-transform infrared spectroscopy (FTIR). The obtained spectra are presented in Figure 12, Figure 13 and Figure 14, respectively.

In all cases—regardless of the substrate type or coating composition—the recorded spectra exhibited a similar pattern of characteristic absorption bands, differing only in signal intensity. This indicated that the chemical nature of the deposited layer remained consistent across the examined range.

The broad band with a maximum around 3330 cm^−1^ corresponded to the stretching vibrations of hydroxyl (O–H) groups and hydrogen-bonded silanol (Si–OH) groups. The bands observed at 3064 cm^−1^ and approximately 2980 cm^−1^ were attributed to C–H stretching vibrations in CH_2_ groups. The absorption band at 1601 cm^−1^ was associated with C=C stretching vibrations in vinyl groups, while the signal at 1408 cm^−1^ corresponded to in-plane bending of CH_2_ groups. The band near 1276 cm^−1^ was related to rocking vibrations of C–H bonds.

The characteristic bands for asymmetric stretching of the Si–O–Si bond were recorded at wavenumbers of 1024 cm^−1^ and 1001 cm^−1^. In the region around 963 cm^−1^, stretching vibrations in the plane of the silanol (Si–OH) groups were visible. The presence of a band at 890 cm^−1^ was attributed to the Si–O–Ti bond, confirming potential chemical interaction between the coating and the titanium substrate. Additionally, at 759 cm^−1^, a signal characteristic of symmetric stretching of the Si–O–Si bonds was recorded. A band around 530 cm^−1^ may have corresponded to twisting vibrations of the vinyl group CH=CH_2_, while the signal at approximately 418 cm^−1^ was related to deformation vibrations of the O–Si–O bond.

The recorded FTIR spectra unequivocally confirmed the presence of key functional groups characteristic of both the organic component (VTMS) and the inorganic component (TEOS), indicating the successful formation of a hybrid polysiloxane matrix [28,30,41,42,43,44,45,46,47,48].

### 3.5. Corrosion Resistance Studies in Ringer’s Solution

**Figure 15:**
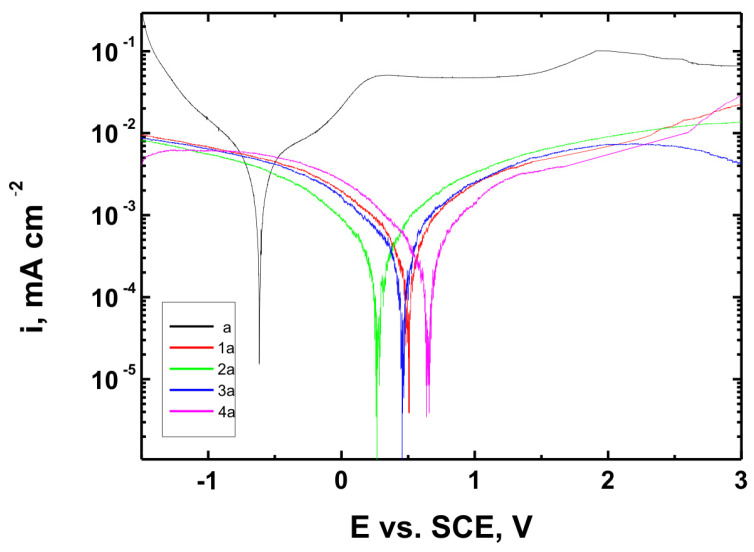
Potentiodynamic polarization curves recorded in Ringer’s solution for Ti Grade 2 uncoated (a) and Ti Grade 2 coated with coatings 1a, 2a, 3a, and 4a.

**Figure 16:**
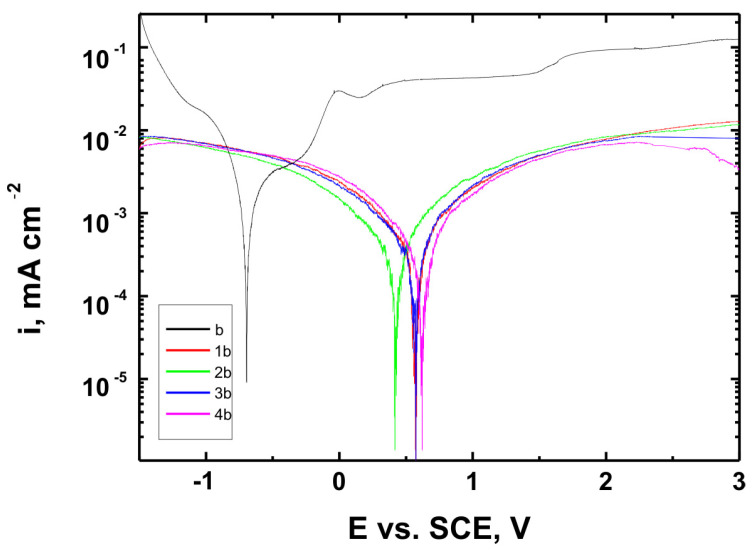
Potentiodynamic polarization curves recorded in Ringer’s solution for Ti6Al4V alloy uncoated (b) and Ti6Al4V alloy coated with coatings 1b, 2b, 3b, and 4b.

**Figure 17:**
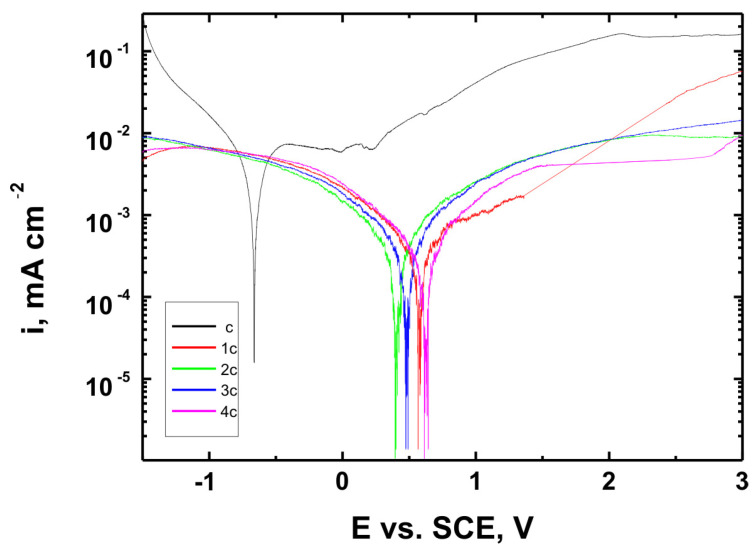
Potentiodynamic polarization curves recorded in Ringer’s solution for Ti13Nb13Zr alloy uncoated (c) and Ti13Nb13Zr alloy coated with coatings 1c, 2c, 3c, and 4c.

**Figure 18:**
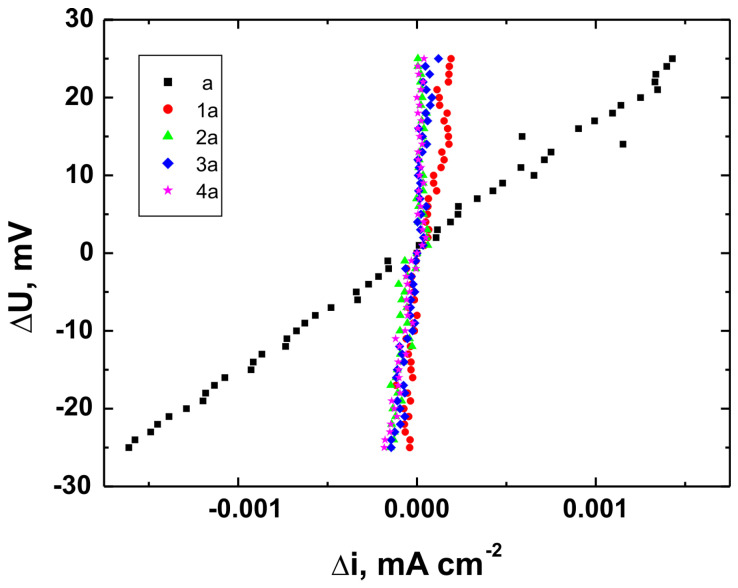
Polarization resistance plots for Ti Grade 2 uncoated (a) and Ti Grade 2 coated with coatings 1a, 2a, 3a, and 4a.

**Figure 19:**
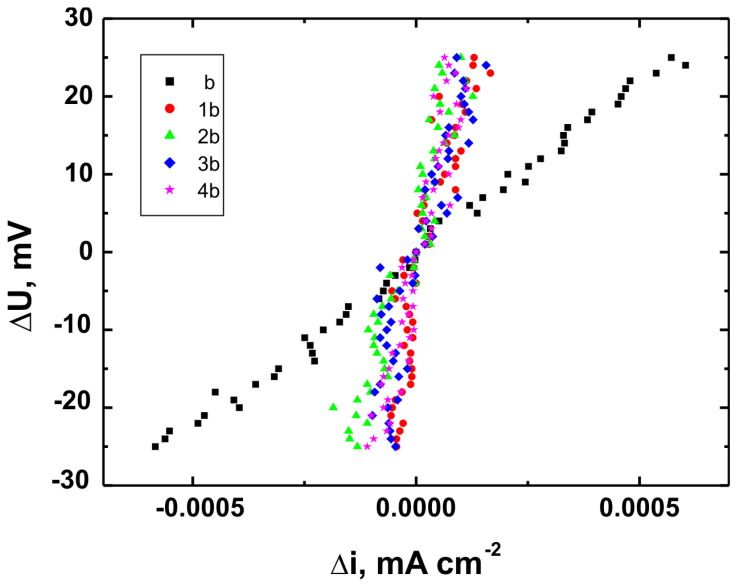
Polarization resistance plots for Ti6Al4V uncoated (b) and Ti6Al4V coated with coatings 1b, 2b, 3b, and 4b.

**Figure 20:**
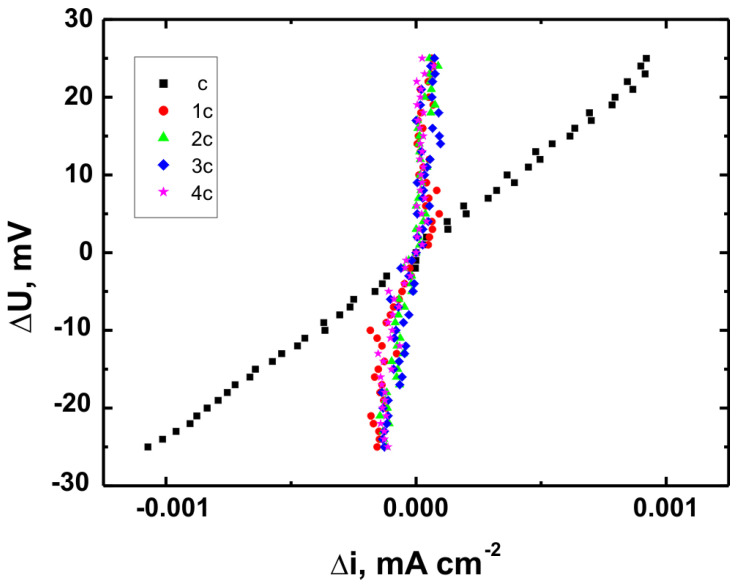
Polarization resistance plots for Ti13Nb13Zr uncoated (c) and Ti13Nb13Zr coated with coatings 1c, 2c, 3c, and 4c.

Figure 15, Figure 16 and Figure 17 present the potentiodynamic polarization curves obtained for coatings deposited on the Ti Grade 2, Ti6Al4V, and Ti13Nb13Zr substrates, respectively, recorded in synthetic physiological fluid—Ringer’s solution. The polarization resistance (Rp) plots, marked with corresponding colors, are shown in Figure 18, Figure 19 and Figure 20.

The corrosion potential (E_corr_ relative to SCE [V]) and polarization resistance (Rp [kΩ·cm^2^]) values are summarized in Table 6. These parameters are key indicators of the protective efficiency of the studied coatings.

Higher Rp values and the shift of the corrosion potential toward more anodic (less negative) values indicated an improvement in corrosion resistance in the presence of VTMS/TEOS coatings compared to uncoated substrates. Particularly favorable values were observed for the 1:1 (VTMS:TEOS) coatings, suggesting an optimal balance between the inorganic and organic components in terms of layer impermeability and adhesion.

The gathered data unequivocally confirmed that the applied coatings significantly enhanced the corrosion resistance of titanium alloys in an environment simulating physiological conditions, making them potentially useful for biomedical applications, especially in implant engineering.

The analysis of parameters derived from potentiodynamic polarization curves confirmed that the best corrosion properties were exhibited by the coatings labeled 4a, 4b, and 4c, in which the molar ratio of VTMS to TEOS was 1:1, regardless of the substrate type (Ti Grade 2, Ti6Al4V, Ti13Nb13Zr). For these samples, the largest shift of the corrosion potential (E_corr_) toward more positive values compared to the reference samples (uncoated substrates) was observed, which clearly indicated an improvement in corrosion resistance in an environment simulating physiological conditions.

The anodic and cathodic current densities were reduced for all coated samples, confirming the effectiveness of the barrier layer. Additionally, for each analyzed system, a significant increase in polarization resistance (Rp) was recorded compared to the uncoated samples, indicating the effective limitation of ion exchange between the substrate and the Ringer’s solution.

The mechanisms of corrosive degradation in hybrid sol–gel coatings are often associated with localized initiation through surface microdefects or porosity in the layer. Over time, aggressive ions such as Cl^−^ present in Ringer’s solution can diffuse through low-density regions of the siloxane matrix, potentially reaching the metal–coating interface. However, in the case of samples 4a–4c, the reduced current densities and increased Rp values suggested a compact, uniform layer with effective barrier properties that limit ion penetration and underfilm corrosion. This supported the hypothesis that an optimized 1:1 precursor ratio leads to a more cross-linked and impermeable siloxane network.

The smallest improvement in corrosion resistance—particularly lower shifts in E_corr_ and lower Rp values—was observed for coatings with a high content of the organic component (VTMS:TEOS = 5:1; samples 2a, 2b, 2c). This may have been associated with a limited polysiloxane network density and greater permeability of these layers to corrosive agents.

In summary, the conducted studies clearly confirmed that the applied hybrid coatings effectively improved the corrosion resistance of titanium and its alloys in an environment simulating bodily fluids. Among all of the analyzed compositions, the most promising application potential in biomaterial engineering was demonstrated by variant 4 (VTMS:TEOS = 1:1), providing the best combination of protective and structural properties. In addition to the electrochemical performance indicators, the long-term stability of the coatings may be affected by localized degradation mechanisms. In chloride-rich environments such as Ringer’s solution, corrosion degradation can potentially initiate via pitting at microdefects or through gradual ion diffusion across the siloxane network. The degree of cross-linking in the hybrid matrix, coating thickness, and interfacial adhesion are crucial factors that determine the coating’s resistance to electrolyte penetration. These aspects depend on the precursor ratio and substrate chemistry, which together determine the durability of the protective barrier under physiological conditions.

The corrosion performance observed in this study compared favorably with data reported in the literature for titanium and titanium alloys coated with sol–gel-derived systems. For example, the polarization resistance (Rp) values for the coated samples in this work exceeded 140 kΩ·cm^2^ in all cases, reaching nearly 240 kΩ·cm^2^ for the best-performing systems. These values were substantially higher than those observed for uncoated titanium substrates (16–44 kΩ·cm^2^) and were consistent with or superior to values reported in the literature for sol–gel-based hybrid or oxide coatings in simulated body fluids [49,50,51,52]. This confirmed the high protective efficiency of the applied VTMS/TEOS coatings. In addition to high Rp values, a significant positive shift in corrosion potential (E_corr_) was observed for all coated samples. For instance, the 4b coating on Ti6Al4V shifted E_corr_ from −0.704 V (uncoated) to +0.622 V, indicating a notable reduction in corrosion susceptibility.

### 3.6. Microstructural Analysis of Coatings After Corrosion Testing in Ringer’s Solution

**Figure 21:**
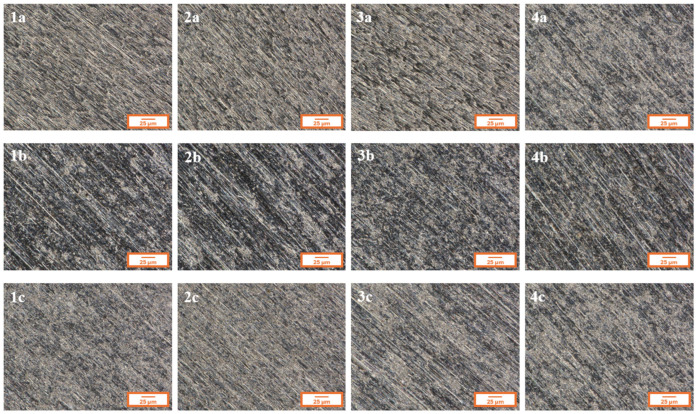
Microstructural images of individual coatings after corrosion testing in Ringer’s solution.

Figure 21 presents microstructural images of selected VTMS/TEOS coatings after corrosion testing conducted in Ringer’s solution. Microscopic surface analysis did not reveal any local corrosion spots, discolorations, pitting, or signs of mechanical degradation such as cracks, peeling, or delamination.

The observed surface conditions indicated high coating stability in a physiological environment, while also confirming their effectiveness as a protective barrier isolating the metallic substrate from the aggressive electrochemical environment. The absence of damage to the coating layer after exposure to Ringer’s solution correlated with the electrochemical measurement results, serving as confirmation of the structural and adhesive integrity of the studied coating systems.

This study focused on the physicochemical analysis and corrosion resistance of VTMS/TEOS hybrid coatings. However, the literature also indicates their high biological potential. As shown in in vitro studies, these coatings exhibit good biocompatibility and lack cytotoxicity toward osteoblast cells [4,53]. Additionally, the silica structures formed as a result of TEOS condensation can serve as bioactive matrices that support the integration of the material with the surrounding bone tissue, as confirmed in in vivo studies, where the presence of such layers initiated new bone tissue formation and supported the osteointegration of implants [54].

In light of these findings, VTMS/TEOS systems can be considered a promising alternative to conventional passive coatings used in implantology, offering both protective and biofunctional properties.

## 4. Summary and Conclusions

In this study, hybrid silane coatings were developed using the sol–gel method, with vinyltrimethoxysilane (VTMS) as the organic precursor and tetraethoxysilane (TEOS) as the inorganic precursor. Four variants of coatings with different VTMS to TEOS ratios were created and applied to three types of titanium substrates, Ti Grade 2, Ti6Al4V, and Ti13Nb13Zr, which are widely used in biomedical applications.

Comprehensive physicochemical characterization of the coatings was performed, including microstructural analysis, 3D surface roughness evaluation, thickness measurements, adhesion tests, and chemical analysis using FTIR. The obtained results clearly confirmed that the produced coatings were continuous, homogeneous, smooth, and free from morphological defects. Their high quality was also confirmed by adhesion tests—all coatings were classified as 4B in the Scotch™ test according to ASTM D3359, indicating excellent adhesion to the metallic surface. However, coatings with a higher TEOS content (series 5–7) showed cracking and delamination, which prevented further analysis, confirming that excessive cross-linking of the inorganic matrix reduced the flexibility and integrity of the coatings.

FTIR spectra obtained for all variants confirmed the presence of key functional groups characteristic of hybrid structures, such as CH_2_, C=C, C–H, Si–O–Si, Si–OH, Si–O–Ti, CH=CH_2_, and O–Si–O. Their presence confirmed the successful hydrolysis and condensation of precursors during the sol–gel process.

The corrosion resistance tests in Ringer’s solution showed that VTMS/TEOS coatings significantly improved the protective properties of all titanium substrates compared to uncoated samples. A decrease in anodic and cathodic current densities and a noticeable increase in polarization resistance (Rp) were observed, which clearly indicated the effective barrier action of the coatings. Coatings from series 4 (VTMS:TEOS = 1:1) were particularly distinguished, achieving the highest protective parameter values—regardless of the substrate type.

Based on the obtained results and literature data, it can be concluded that the developed VTMS/TEOS hybrid coatings represent a promising solution in biomaterial surface engineering, particularly in the context of implant applications, where it is crucial to limit corrosion processes and ensure long-term stability of implants. An important advantage of these coatings is also their potential bioactivity and biocompatibility, as described in the literature—including the absence of cytotoxicity toward osteoblasts and their promotion of integration with bone tissue.

In summary, the VTMS/TEOS coating with a 1:1 ratio showed the greatest application potential as a passivation layer for titanium implants, effectively minimizing the risk of corrosion and thereby contributing to the improvement of clinical safety for long-term implants.

In the context of the literature, the presented research makes a significant contribution to the development of sol–gel technology for biomedical applications. Unlike previous studies that primarily used highly acidic catalysts (e.g., HNO_3_) and non-biocompatible substrates (such as steel or tin), this work is the first to introduce the use of acetic acid as a hydrolysis catalyst in the VTMS/TEOS system applied to medical-grade titanium substrates. Additionally, the selection of three distinct titanium alloys enabled a comprehensive evaluation of the coatings’ effectiveness in terms of adhesion, corrosion resistance, and potential bioactivity. This approach has not been previously described and opens up new possibilities for designing functional, biocompatible protective systems for long-term implants.

In the next stages of research, it is planned to expand the analysis by assessing biocompatibility using in vitro methods, particularly to determine the impact of the coatings on the adhesion, proliferation, and differentiation of osteoblastic cells, in order to confirm their full potential for clinical applications in implantology.

Theoretical and mechanical studies—such as reaction kinetics, thermodynamics, DFT modeling, and high-resolution XPS analysis—represent promising directions for future research to complement the experimental findings presented here.

## Figures and Tables

**Table 1:** Chemical compositions of Titanium Grade 2, Ti6Al4V alloy, and Ti13Nb13Zr alloy.

	Ti Grade 2	Ti6Al4V	Ti13Nb13Zr
Fe	max. 0.30	max. 0.30	max. 0.25
O	max. 0.25	max. 0.20	max. 0.15
C	max. 0.08	max. 0.08	max. 0.08
N	max. 0.03	max. 0.05	max. 0.05
H	max. 0.015	max. 0.0125	max. 0.012
Y	—	max. 0.005	—
Al	—	5.50–6.75	—
V	—	3.50–4.50	—
Nb	—	—	12.5–14.0
Zr	—	—	12.5–14.0
Ti	base	base	base

**Table 2:** Three-dimensional surface roughness parameters of the coatings on the Ti Grade 2 substrate.

Sample	1a	2a	3a	4a
Sa [μm]	0.27 ± 0.03	0.31 ± 0.02	0.30 ± 0.02	0.30 ± 0.00
Sz [μm]	3.46 ± 0.92	2.72 ± 0.24	2.57 ± 0.31	2.47 ± 0.29
Sq [μm]	0.34 ± 0.03	0.37 ± 0.03	0.37 ± 0.03	0.37 ± 0.01
Ssk	0.66 ± 0.10	0.45 ± 0.07	0.58 ± 0.19	0.59 ± 0.16
Sku	3.73 ± 0.21	2.83 ± 0.28	3.25 ± 0.47	3.08 ± 0.49
Sp [μm]	2.50 ± 0.99	1.71 ± 0.23	1.57 ± 0.27	1.61 ± 0.31
Sv [μm]	0.96 ± 0.12	1.01 ± 0.04	1.00 ± 0.08	0.86 ± 0.04

**Table 3:** Three-dimensional surface roughness parameters of the coatings on the Ti6Al4V substrate.

Sample	1b	2b	3b	4b
Sa [μm]	0.26 ± 0.01	0.28 ± 0.03	0.29 ± 0.02	0.27 ± 0.01
Sz [μm]	2.41 ± 0.34	2.16 ± 0.17	2.28 ± 0.25	1.95 ± 0.17
Sq [μm]	0.32 ± 0.02	0.35 ± 0.03	0.36 ± 0.02	0.33 ± 0.02
Ssk	0.76 ± 0.16	0.53 ± 0.04	0.72 ± 0.09	0.66 ± 0.09
Sku	3.43 ± 0.54	2.78 ± 0.11	3.12 ± 0.15	2.89 ± 0.28
Sp [μm]	1.39 ± 0.14	1.33 ± 0.12	1.56 ± 0.22	1.21 ± 0.14
Sv [μm]	1.02 ± 0.27	0.84 ± 0.04	0.73 ± 0.03	0.73 ± 0.05

**Table 4:** Three-dimensional surface roughness parameters of the coatings on the Ti13Nb13Zr substrate.

Sample	1c	2c	3c	4c
Sa [μm]	0.30 ± 0.03	0.27 ± 0.03	0.30 ± 0.02	0.29 ± 0.02
Sz [μm]	3.16 ± 0.87	2.73 ± 0.71	2.63 ± 0.44	2.40 ± 0.40
Sq [μm]	0.37 ± 0.02	0.33 ± 0.04	0.38 ± 0.02	0.35 ± 0.03
Ssk	1.00 ± 0.53	0.71 ± 0.19	0.65 ± 0.15	0.61 ± 0.03
Sku	6.20 ± 4.49	4.13 ± 1.51	3.17 ± 0.45	2.95 ± 0.20
Sp [μm]	2.29 ± 0.97	1.93 ± 0.67	1.77 ± 0.45	1.60 ± 0.43
Sv [μm]	0.88 ± 0.10	0.80 ± 0.07	0.85 ± 0.09	0.80 ± 0.10

**Table 5:** Average thickness of individual coatings with measurement uncertainties. Values are given as the mean ± standard deviation (SD) based on ten independent measurements taken at different sample regions.

Coating	Thickness [μm]
1a	14.40 ± 3.58
2a	12.52 ± 2.64
3a	13.25 ± 1.53
4a	12.89 ± 1.71
1b	13.32 ± 2.87
2b	14.91 ± 0.72
3b	13.72 ± 1.06
4b	14.31 ± 1.26
1c	11.89 ± 4.26
2c	10.44 ± 1.79
3c	10.93 ± 2.19
4c	10.42 ± 0.95

**Table 6:** Corrosion potential (E vs. SCE [V]) and polarization resistance (Rp [kΩ·cm^2^]) values for the individual samples.

Sample	E vs. SCE [V]	Rp [kΩ·cm^2^]
Ti Grade 2 (a)	−0.625	16.01 ± 0.18
1a	0.511	153.75 ± 8.49
2a	0.260	193.30 ± 16.01
3a	0.455	215.49 ± 12.21
4a	0.638	201.61 ± 15.17
Ti6Al4V (b)	−0.704	44.44 ± 0.53
1b	0.574	223.01 ± 15.47
2b	0.418	180.22 ± 8.99
3b	0.574	185.81 ± 12.30
4b	0.622	239.71 ± 12.22
Ti13Nb13Zr (c)	−0.662	24.70 ± 0.18
1c	0.567	147.60 ± 11.37
2c	0.395	223.57 ± 10.73
3c	0.475	195.45 ± 12.15
4c	0.613	198.51 ± 13.25

## Data Availability

The original contributions presented in this study are included in the article. Further inquiries can be directed to the corresponding authors.
